# Characterizing pancreatic cancer-associated fibroblast heterogeneity in vascular microphysiological systems

**DOI:** 10.1007/s10456-026-10061-9

**Published:** 2026-06-09

**Authors:** Charles W. Blackledge, Sarah Gullion, Ian McCabe, Ngan N. K. Van, Arthi Hariharan, Changfei Luan, Xianlu Laura Peng, William J. Polacheck, Jen Jen Yeh, Sarah E. Shelton

**Affiliations:** 1 Lampe Joint Department of Biomedical Engineering, University of North Carolina and North Carolina State University, Raleigh, NC USA; 2 Wilson College of Textiles, North Carolina State University, Raleigh, NC USA; 3 Department of Cell Biology and Physiology, University of North Carolina, Chapel Hill, NC USA; 4 Lineberger Comprehensive Cancer Center, University of North Carolina, Chapel Hill, NC USA; 5 Department of Pharmacology, University of North Carolina, Chapel Hill, NC USA; 6 Department of Surgery, University of North Carolina, Chapel Hill, NC USA

**Keywords:** Cancer-associated fibroblast, Pancreatic cancer, Vasculature, Microfluidics, Angiogenesis

## Abstract

Pancreatic Ductal Adenocarcinoma (PDAC), one of the deadliest cancer types, is dominated by a tumor microenvironment (TME) that is desmoplastic, immunosuppressive, and generally unresponsive to conventional cancer treatments. Cancer-Associated Fibroblasts (CAFs) are thought to drive many of the features of PDAC that contribute to treatment resistance, yet CAFs exhibit wide phenotypic heterogeneity with unclear biological and clinical implications. Recent work has identified CAF subtypes – tumor-restraining CAFs (RestCAFs) or tumor-promoting CAFs (ProCAFs) – that correlate with overall survival. Therefore, this study aims to identify CAF subtype-dependent functional effects in the TME using 3D microphysiological systems, focusing primarily on their interactions with vasculature. Microfluidic devices including a RestCAF line demonstrated increased levels of vasculogenesis and extracellular matrix remodeling, while devices containing a ProCAF line exhibited increased angiogenic sprouting and differential immune cell recruitment, such as monocyte migration into the TME. This work provides novel insights into the heterogeneous functions of CAFs in the TME and lays important groundwork for the development of CAF-targeted therapies in PDAC.

**Supplementary Information:**

The online version contains supplementary material available at 10.1007/s10456-026-10061-9.

## Introduction

Pancreatic Ductal Adenocarcinoma (PDAC) is the dominant form of pancreatic cancer, representing roughly 90% of all pancreatic cancer diagnoses [1]. PDAC is the 10th most common cancer type with an estimated 67,440 cases in 2025, and is expected to be the 3rd most deadly cancer with 51,980 deaths [2]. Physiologically, PDAC is characterized by a highly desmoplastic and heterogeneous tumor microenvironment (TME) [3], with abundant extracellular matrix (ECM), fibroblasts, immune cells, and secreted factors dominating the space surrounding the tumor. This dense TME may dramatically increase resistance to conventional chemotherapy and immunotherapy treatment regimens, leading to tumor growth and metastasis [3, 4]. Furthermore, only 15% of tumors are diagnosed before metastasis [2], and 75% of stage 0 and stage I patients display no symptoms upon diagnosis [5, 6]. This suggests that PDAC can grow undetected for extended periods of time and explains the dismal 5-year survival rate of 13% for patients with PDAC [7].

A major cellular component of the PDAC TME is fibroblasts - the primary ECM-producing cell and one of the most dynamic cell types in the body [4]. Fibroblasts are present in tissues throughout the body and have indispensable roles in the wound healing response. Quiescent stromal fibroblasts are activated by pro-inflammatory cytokines in response to injury [8], and are involved in stimulating inflammation [9], reconstructing stroma [10], and recruiting immune cells [11]. Some fibroblasts are able to further differentiate into myofibroblasts, which express alpha-smooth muscle actin (α-SMA) and are responsible for physically closing wounds [12]. Appropriately, fibroblasts are referred to as “choreographers of the wound healing response” [13]. Once wound healing has concluded, activated stromal fibroblasts and myofibroblasts often revert to a quiescent state or undergo apoptosis [4], but persistent phenotypic changes often occur in the context of cancer. Since tumors are often defined as “nonhealing wounds” due to large amounts of chronic fibrosis and inflammation [14], fibroblasts near tumors often continuously produce dense stroma, and fail to inactivate or undergo apoptosis [4, 15]. These types of fibroblasts are defined as Cancer-Associated Fibroblasts (CAFs), and are the main contributors to the highly desmoplastic nature of the PDAC TME [4, 16].

CAFs were originally defined as α-SMA-expressing fibroblasts within solid tumors [15, 17], but research has identified numerous CAF subtypes with opposing phenotypes [18], suggesting a high degree of CAF heterogeneity. Commonly described subtypes include myofibroblastic CAFs (myCAFs) and inflammatory CAFs (iCAFs). MyCAFs express α-SMA and are highly contractile [19], whereas iCAFs express little α-SMA, and secrete elevated levels of cytokines such as IL-6 and CXCL12. While iCAFs have been thought to exert more control over the immune response, in fact, both myCAFs and iCAFs have shown immunosuppressive properties via limiting T-cell recruitment [20–22]. Ultimately, the stroma-producing and immune-evading abilities of CAFs result in a PDAC TME that is highly resistant to conventional cancer treatments, dramatically worsening the clinical outlook of patients with PDAC [22, 23].

Due to the influence of CAFs in the PDAC TME, considerable effort has been made to identify new PDAC treatment methods by targeting CAFs. However, in vivo studies that have attempted to target myCAFs and iCAFs by depleting CAF-derived ECM [24, 25], inhibiting tumor-CAF signaling [26, 27], and modifying CAF-immune cell interactions [26, 28] have had mixed results in treating PDAC. This limited efficacy highlights our incomplete understanding of CAF heterogeneity and physiological functions in the PDAC TME. Further, the use of animal models to study CAF-targeted treatments results in studies that both fail to accurately replicate the human PDAC TME and produce results that are difficult to translate into clinical treatments. To address these problems, microphysiological systems (MPS) have been developed by combining microfluidic device technology with human cells to mimic in vivo tissue structures [29]. MPSs offer the ability to study the PDAC TME in 3D environments with higher levels of physiological relevance compared to 2D cell culture methods [30] and increased clinical relevance of treatments compared to animal studies [31]. Thus, MPSs serve as viable platforms to study complex cellular and tissue functions in vitro.

Peng et al. recently identified two distinct CAF subtypes in PDAC that correlate with overall patient survival [25]. Using single-cell RNA sequencing with the SCISSORS approach, CAF clusters of similar cell phenotypes were generated [32]. By comparing these clusters to the clinical outcomes of patients, two notable subtypes of CAF emerged: “tumor-restraining” (RestCAFs) and “tumor-promoting” (ProCAFs) [25]. Single-cell RNAseq analysis of PDAC tissue identified significant overlap between the established iCAF/myCAF subtypes and the novel RestCAF/ProCAF subtypes respectively, where 85.2% of iCAF tumors were classified as RestCAF, and 62.6% of myCAF tumors were classified as ProCAF [25]. In addition, mean overall survival rates (mOS) of patients with RestCAF tumors (mOS 29.04 months) are significantly longer than patients with ProCAF tumors (mOS 17.70 months) – hinting at the presence of potential CAF-mediated biological impacts within the PDAC TME [25].

Though the stratification of patients by RestCAF or ProCAF subtype strongly correlates with overall survival, we do not yet know how CAFs exert these effects in the PDAC TME. Therefore, in this study, we have revealed key physiological differences between two distinct CAF subtypes utilizing 3D microfluidic models of a vascularized PDAC TME. We identified differences in secreted factor expression, contractility in 3D ECM, vasculogenesis, endothelial permeability (barrier function), angiogenesis, and immune cell recruitment between one representative RestCAF line and one representative ProCAF line. These findings provide both novel insight into the mystery of CAF heterogeneity and a platform for testing the development of CAF-targeted treatments.

## Materials and methods

### CAF isolation and subtype assignment

Primary CAF cell lines were derived from de-identified patient tumors under an approved IRB exemption using the outgrowth method. Tumors were minced into pieces no larger than 1 mm^3^ and cultured in Advanced DMEM/F-12 (Gibco #12634010) supplemented with 15% fetal bovine serum (FBS) (Sigma #F2442-500ML), 1x glutamine (Gibco #35050079), 1x HEPES (Corning #25-060-CI). Before the fifth passage, CAF lines were immortalized using human telomerase reverse transcriptase (h-TERT, Addgene#1771). After immortalization, CAFs were cultured in “CAF medium” (Advanced DMEM/F-12 supplemented with 8% FBS, 1x glutamine, 1x HEPES, and 1% penicillin/streptomycin antibiotic [GenClone, 25–512]).

Each sample was defined as the RestCAF or ProCAF subtype using the DeCAF classifier to assign subtype probability scores based on expression of key genes as described in Peng et al. [25]. Briefly, RNA was extracted and libraries were generated, followed by sequencing [25]. Raw sequencing files in BCL format were converted to fastq files using bcl2fastq2 (v2.19.0). RefSeq assembly (GCF_000001405.40) of the human reference genome GRCh38.p14 was used as the reference for gene quantification by Salmon 1.9.0 (“-- gcBias -- seqBias”) [33]. The total expected read counts per gene were normalized to transcripts per million (TPM). One RestCAF line (DeCAF score = 0.034368) and one ProCAF line (DeCAF score = 0. 94570233) were selected and used throughout this study.

### Cell culture

Human Umbilical Vein Endothelial Cells (HUVEC) expressing Green Fluorescent Protein (GFP) and immortalized with h-TERT were cultured using flasks coated with 0.5% gelatin in Vasculife VEGF Complete Endothelial Medium with 0.19 U/ml heparin sulfate (LifeLine Cell Technologies, SKU# LL-0003). CAFs were cultured in CAF medium. Cells were maintained in an incubator at 37 °C with 5% CO_2_, and media was changed every 2–3 days. CAFs and HUVECs were grown to 80% confluency prior to incorporation into the devices or passaging. To generate the CAF-derived conditioned media, 8 × 10^5^ RestCAFs or ProCAFs were cultured in 2D with 15 ml of CAF medium for 3 days. The culture media was then removed, sterile filtered to remove any cellular debris, and stored in 1mL aliquots at −80 °C.

THP-1 monocyte and Jurkat T cell lines were grown in suspension in RPMI 1640 medium (Corning #10-040-CV) medium supplemented with 10% FBS and 1% penicillin/streptomycin. Cells were maintained in an incubator at 37 °C with 5% CO_2_ for 2 days prior to incorporation in experiments. HL-60 (ATCC) cells were differentiated into a neutrophil-like state by suspending them at 0.3 × 10^6^ cells/mL in RPMI supplemented with 10% FBS and 1% penicillin/streptomycin plus 1.3% dimethyl sulfoxide (DMSO, Ward’s Science #470300-982). Cells undergoing differentiation were maintained in an incubator at 37 °C with 5% CO_2_ and left undisturbed for 5 days to allow the development of a neutrophil-like phenotype (nHL60). Cryopreserved human peripheral blood mononuclear cells (PBMCs) were obtained from HumanCells Biosciences from a healthy donor (male, 38) and thawed immediately prior to use.

### Microfluidic device fabrication

#### General fabrication methods

Microfluidic devices were fabricated in polydimethylsiloxane (PDMS, Sylgard 184) using a 10:1 ratio of the base to curing agent. PDMS was degassed and cured overnight at 65 °C. Individual devices were cut out using a razor blade, media inlet ports were created with a 4 mm biopsy punch, and ECM inlet ports were created with a 1 or 1.5 mm biopsy punch. Devices were then plasma-bonded to a No. 1.5 glass coverslip.

#### 3-channel microfluidic devices

A 3-channel microfluidic device was utilized for vasculogenesis and CAF constriction experiments. The device consists of a center ECM channel, 3 mm wide, and two media channels flanking the ECM channel. Channels are 500 μm high and delineated by 150 μm phase guides. The master mold was designed using AutoDesk Fusion 360 and CAM milled in polyoxymethylene (Delrin, McMaster Carr) using a CNC Desktop Milling Machine (Bantam Tools). After device fabrication and plasma bonding, devices were filled with 70% ethanol and placed in a 65 °C oven for 48 h to sterilize and restore hydrophobicity. 

#### Single-vessel microfluidic devices

A single-vessel microfluidic device was utilized for immune cell migration and sprouting angiogenesis experiments. This device has a central ECM region bisected by a long, thin needle guide leading to two media ports. The master negative mold was fabricated using photolithography, as described in Polacheck et al. [34]. After device fabrication and plasma bonding, the devices were filled with a solution of 2 mg/mL dopamine hydrochloride (Millipore Sigma #H8502-5G) dissolved in 10 mM Tris Hydrochloride buffer, ph 8.5 (Teknova #T5085) for 1 h in darkness, followed by 2 washes with sterile water.

Next, a 0.01% solution of bovine serum albumin (BSA, Millipore Sigma #A7030) in phosphate buffered saline (PBS) was prepared and sterilized through a 0.22 μm filter. Sterile stainless steel needles (160 μm diameter) were soaked in the BSA solution for 30 min, before being inserted into the device - through the needle guide and bisecting the central region. Each device was then filled with 70% ethanol and UV sterilized for 15 min (B1450 UV Clave, Benchmark Scientific). After sterilization, the remaining ethanol within the devices was aspirated to prepare the devices for cell culture.

### Experimental methods

#### TGF-β1 ELISA

The Quantikine ELISA Human/Mouse/Rat/Porcine/Canine TGF-β1 ELISA kit (R&D Scientific # DB100C) was utilized to measure the amount of TGF-β1 present in RestCAF/ProCAF conditioned media. The assay was conducted in accordance with the manufacturer’s instructions. At the conclusion of the assay, optical densities were measured using a SpectraMax i3x Multi-Mode Microplate Reader.

#### Multiplex CAF conditioned media analysis

ProcartaPlex Human Mix & Match Multiplex Panels (ThermoFisher Scientific) were utilized to measure the concentrations of several secreted factors present in RestCAF and ProCAF conditioned media. Custom panels included targets relevant to angiogenesis and immune cell recruitment. Assays were conducted in accordance with the manufacturer’s instructions for cell-culture supernatant, and CAF conditioned media and growth media were not diluted before testing. At the conclusion of the assays, mean fluorescent intensities and concentrations of targets were measured using a Luminex 200 Instrument System.

#### Vasculogenesis in 3-channel microfluidic devices

To generate microvascular networks (MVNs) via self-assembly, HUVEC and CAF were embedded in a fibrin ECM. Fibrin was selected as the ECM for these experiments due to the existence of many robust protocols for MVN assembly in fibrin, simplifying experimental methods [35, 36]. To begin, thrombin was dissolved at 100 U/mL and stored at -80 °C until further dilution in endothelial medium to a concentration of 4 U/mL immediately prior to use. Upon reaching 80% confluency, HUVECs and CAFs were detached, counted, and resuspended in thrombin to concentrations of 28$\:\times\:$10^6^ cells/mL and 2$\:\times\:$10^6^ cells/mL, respectively. HUVECs and RestCAF/ProCAF cell suspensions were mixed in equal volumes, followed by the addition of 6 mg/mL bovine fibrinogen (Millipore Sigma #F8630-1G) to the cell mixture at a 1:1 ratio. The HUVEC/CAF/fibrinogen mixture was pipetted into the central channel of the 3-channel microfluidic device. Devices were incubated for 15 min to allow for fibrin polymerization, followed by the addition of warm endothelial medium to the media channels. Media was replaced daily for 7 days to allow for the self-assembly of HUVECs into MVNs. To identify perfusable MVNs, a 70 kD fluorescent dextran was perfused through each MVN. Maximum projection confocal images of GFP-HUVEC MVNs and fluorescent dextran within perfusable vessels were captured using a STELLARIS 5 confocal microscope (Lecia Microsystems, Wetzlar, Germany, 2023) and 5x/10x objectives.

#### CAF-mediated fibrin constriction in 3-channel microfluidic devices

To assess the ability for CAFs to constrict a 3D fibrin matrix, we embedded CAFs in a fluorescent fibrin ECM, using the same concentrations of fibrin as used in vasculogenesis experiments. To begin, CAFs were labeled with CellTracker Green CMFDA (Table S1) for 20 min, washed, and resuspended to a concentration of 4$\:\times\:$10^6^/mL in 4U/mL thrombin. 6 µL of each CAF/thrombin suspension was mixed with 6 µL of far-red fluorescent fibrinogen - generated by spiking 2% by volume of 2.5 mg/ml AlexaFluor 647 fibrinogen from human plasma (Fisher Scientific #F35200) in 6 mg/mL bovine fibrinogen. The cell suspension was loaded in the central region of the 3-channel microfluidic device at final concentrations of 2 × 10^6^, 1 × 10^6^, and 0.5 × 10^6^ cells/mL. The devices were placed in an incubator for 15 min to allow the fibrin to polymerize prior to filling the media channels with Vasculife medium. Devices were cultured for 1 week with daily media changes and then imaged via confocal microscopy.

#### Single-vessel microfluidic device preparation

Single-vessel models were generated by filling microfluidic devices containing a stainless-steel needle with CAFs embedded in collagen, followed by applying an endothelial monolayer to the cylindrical lumen left after removing the needle. Collagen was used as the ECM inside this model to replicate the existing methodology by Polacheck et al. [34]. Briefly, upon reaching approximately 80% confluency, CAFs were dyed with a CellTracker fluorescent probe diluted in PBS to enable live-cell imaging (Table S1). RestCAFs and ProCAFs were incubated with the desired fluorescent dye for 20 min. After 2 washes in FACS buffer (PBS + 2% FBS), CAFs were detached, counted, and resuspended to 1$\:\times\:$10^6^ cells/mL. Meanwhile, 600 µL of a 2 mg/mL Type 1 Collagen solution was prepared (Table S2). All reagents were kept on ice, and collagen was mixed thoroughly until a pale orange color was achieved, indicating neutral pH. Care was taken while mixing to minimize the introduction of bubbles into the collagen solution. Next, 1 × 10^5^ RestCAFs and ProCAFs were separated, centrifuged, and resuspended into 200 µL of collagen each, to create a suspension of 5 × 10^5^ cells/mL of each CAF type in collagen. The center ECM region of each device was then gently filled with 25 µL of these suspensions. The collagen was allowed to polymerize inside the device for 30 min in an incubator. After collagen polymerization, each media reservoir was filled with CAF medium, and a small drop of medium was placed on each ECM port. Devices were incubated overnight before gently removing the needle and sealing the needle guide with vacuum grease pre-sterilized via autoclaving (Molykote, Millipore Sigma #Z273554). Devices were then filled with Vasculife and incubated overnight before coating the channels with endothelial cells.

To add an endothelial monolayer to the interior surface of the lumen created by removing the needle, GFP-HUVECs were detached, counted, and resuspended to a concentration of 1 × 10^6^ cells/mL in Vasculife. All culture media was then aspirated from the microfluidic devices, and the HUVEC cell suspension was added to the media reservoirs with 60 µL on one side and 30 µL on the other side to produce flow through the channel. Each device was rotated for 5 minutes with the channel parallel to the axis of rotation at 5 rpm and 37 °C (Roto-Therm Plus Incubated Rotator, Benchmark Scientific), ensuring HUVECs formed even layer around the circumference of the channel. After a consistent cell density was achieved, the cell suspension was removed from both media reservoirs simultaneously with a multichannel pipette, and the device was refilled with fresh Vasculife, refreshed daily as needed. The vessels were cultured for at least one day before testing vessel permeability and immune cell extravasation to ensure the formation of a confluent monolayer.

#### Single-vessel permeability analysis

Vascular permeability of the single-vessel microfluidic model was assessed one day after a successful HUVEC coating by capturing time-lapse images of fluorescent dextran diffusion into the collagen ECM. We used three molecular weights of dextran: 3,000 Da, 10,000 Da, and 70,000 Da to obtain vessel permeability coefficients as functions of time and molecular weight. Vessel permeability protocols were adapted from Polacheck et al. [34]. One day after a successful HUVEC coating, dextran solutions (Table S3) were diluted in Vasculife to a concentration of 0.1 mg/ml. Working with one device at a time, existing Vasculife inside the device was removed, and one media port was filled with 50 µL of the diluted dextran solution. Confocal images were then immediately captured at the midplane of the vessel every 10 s for 2 minutes. Images were captured with a STELLARIS 5 Confocal Microscope and a 10x objective. Fluorescent intensity of dextran within collagen and vessel permeability coefficients were calculated using MATLAB code provided by Polacheck et al. [34].

#### Immune cell recruitment in single-vessel models

Differential recruitment of immune cells by RestCAFs and ProCAFs were investigated by flowing immune cells through the single-vessel microfluidic model and quantifying both the number of extravasated immune cells and the migration distance through the collagen. We used THP-1 (monocytic leukemia) to represent monocyte/myeloid cell recruitment, Jurkat cells (T cell leukemia) to represent T cell recruitment, and differentiated HL-60 cells (promyelocytic leukemia) to represent neutrophil recruitment. In addition, we used primary PBMCs from a healthy donor to validate cell line data and increase clinical translatability of our findings. One day after successful HUVEC coating, immune cells (monocytes, T cells, neutrophils, or PBMCs) were dyed with a CellTracker fluorescent probe diluted in PBS (Table S1). The immune cells were incubated with the fluorescent probe for 20 min, washed 3 times in PBS + 2% FBS, and resuspended to 1 × 10^6^ cells/mL in Vasculife. All media was removed from each media reservoir and collagen port. To enable both a higher flow rate and a prolonged period of flow through the device, a 1 mL syringe cap was inserted into one media reservoir. This syringe cap was filled with 100 uL of the immune cell suspension, and the opposite media reservoir was filled with 30 uL of medium. This pressure difference resulted in an approximate mean flow velocity of 4.018 mm/sec and a mean wall shear stress of 2.012 dyne/cm^2^ – reflecting physiological values for low-pressure human vasculature [37]. These values were estimated by flowing fluorescent beads through the single-vessel model and tracking bead position over time with ImageJ. A video of fluorescent bead flow is provided as Supplementary Movie S1.

After verification of immune cell flow under a brightfield microscope, the device was incubated for 30 min on a rocker, with the vessel parallel to the rocking axis. The combination of pressure-driven flow and orthogonal rocking motion aims to discourage the settling of immune cells on the bottom of the vessel caused by the gradual equalization of the pressure gradient that reduced the flow rate over time. After 30 min of flow, the syringe cap, cell suspension, and media were removed, and the device was re-filled with Vasculife and incubated for 2 days at 37 °C and 5% CO_2_. After 2 days of static culture, images of immune cell migration were captured via confocal microscopy.

#### Immunofluorescent staining

Devices were fixed in 4% paraformaldehyde (PFA), incubated for 15 min at room temperature, and washed 3 times in PBS. Cell membranes were permeabilized for staining of intracellular targets with 0.1% Triton X-100 (ThermoFisher Scientific #A16046.AE) for 10 min. After 2 additional washes in PBS, blocking buffer (5% BSA/PBS + 3% goat serum) was added to the device, and incubated overnight at 4 °C. Primary antibodies were diluted in PBS (Table S4) and incubated overnight at 4 °C. After 5 washes in wash buffer (0.5% BSA/PBS), secondary antibodies were diluted in PBS (Table S4) and incubated overnight at 4 °C. Devices were washed an additional 3 times in wash buffer and twice in PBS before imaging. Ulex Europaeus Agglutin 1 (UEA-1) FITC Lectin was added to the single-vessel devices for 20 min to visualize the endothelium (Table S5). Lectin was washed twice in medium before imaging. Images were captured using a STELLARIS 5 Confocal Microscope (Lecia Microsystems, Wetzlar, Germany, 2023) with a 5x or 10x objective.

### Image and statistical analyses

#### Microvascular network permeability and geometry analysis

The AutoTube vessel analysis software [38] within Matlab (R2024A) was utilized to analyze maximum projection MVN images for the calculation of perfusion percentages and average vessel widths. Both GFP-HUVEC and fluorescent dextran images were analyzed with AutoTube, and output parameters for each image include the area covered by vessels and the average vessel width. Perfusion percentages were determined by dividing the vessel area of a dextran image by the vessel area of the corresponding GFP image, for each device and CAF type.

#### Fibrin constriction and intensity calculations

Fibrin constriction was calculated from ImageJ using maximum intensity projection images taken after 7 days of culture. A polygon was drawn around the constricted ECM/CAF region, and the percent constricted was calculated by comparing this constriction area to the full area of the central ECM region of the 3-channel microfluidic device. Fibrin intensity values were also calculated from maximum intensity projections in ImageJ. The ECM region of the 3-channel microfluidic device was selected, and the average pixel intensity value of fibrin was measured across this region.

#### Angiogenesis analysis

Angiogenic sprout lengths were quantified using ImageJ. Using maximum projection confocal images captured 3 days after HUVEC coating, the center of each angiogenic sprout was traced manually. The total number and lengths of each sprout were measured in each device, for each CAF type.

#### Single-vessel diameter measurements

Diameters of the single-vessel microfluidic models were quantified using ImageJ. Using maximum projection confocal images captured 3 days after HUVEC coating, three lines were drawn across the vessel on the left, right, and center. The minimum and maximum vessel diameters were included in the measurements. Averaging these measurements resulted in the mean vessel diameter value per device, which was used for statistical analysis.

#### Immune cell recruitment analysis

The number of extravasated immune cells and the migration distance were both analyzed using ImageJ. After obtaining X-Y maximum projection images of each single-vessel device, the perpendicular distance from the vessel wall to the center of each immune cell was measured in ImageJ. To ensure that only cells that extravasated through the endothelium were counted and to eliminate cases of immune cells simply leaking out of holes in the endothelium, extravasated immune cells were not counted if they were near the extreme ends of the vessel or if they were co-localized with the X-Y projection of the endothelium.

#### Statistical analysis

One-Way ANOVA with Tukey’s HSD post-hoc tests, Two-Way ANOVA with Tukey’s HSD post-hoc tests, unpaired T-tests corrected for multiple comparisons with the Holm-Šídák method, and Chi Square Independence Tests were used to determine statistical significance in experiments. Statistical tests were conducted with GraphPad Prism Version 10.4.2. *P* < 0.05 was considered statistically significant. Data comes from 2 or more independent experiments for each assay.

## Results

### Establishing CAF subtype biology with immunofluorescent staining and analyzing secreted factors

**Fig. 1:**
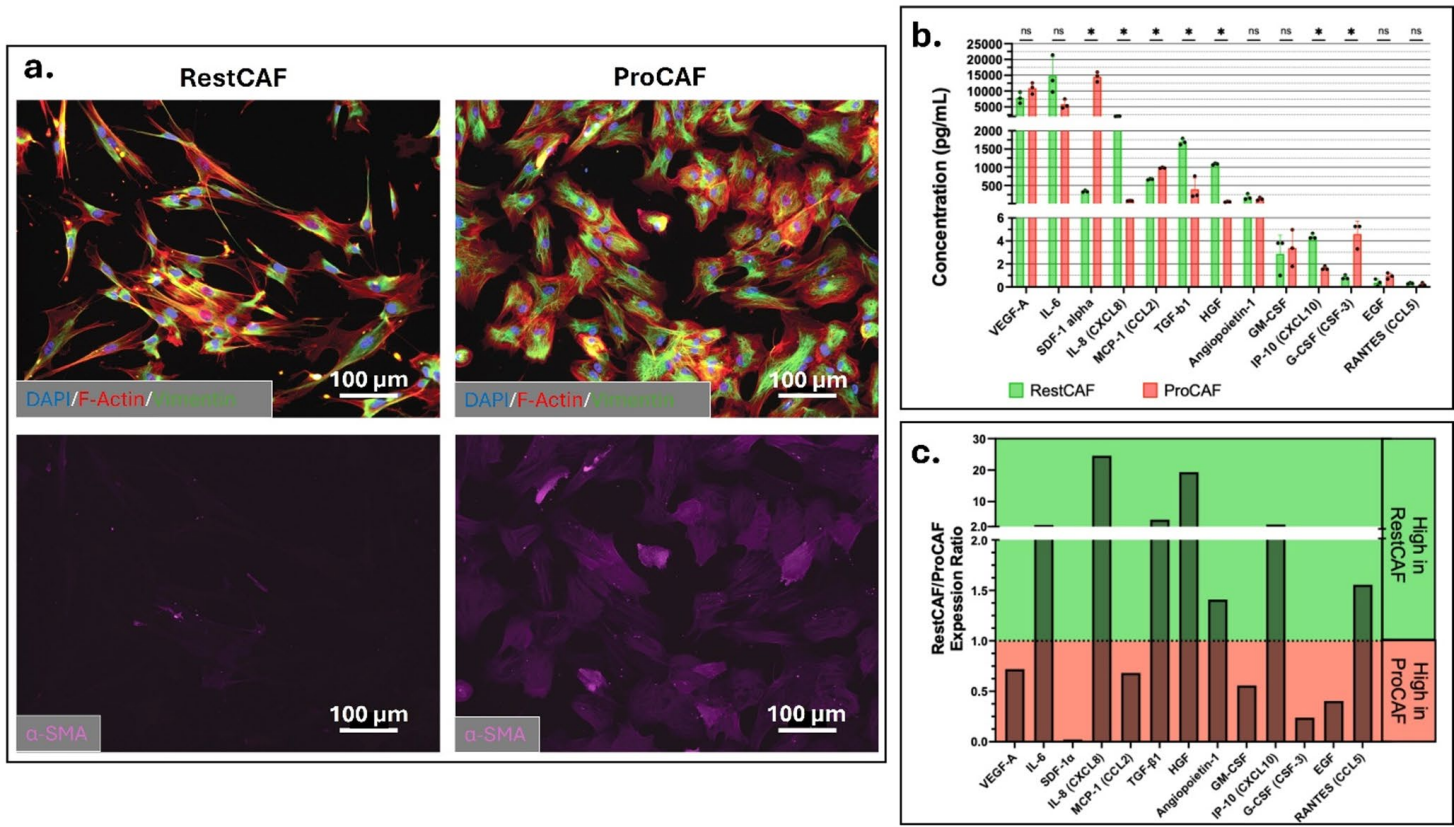
Establishing CAF Subtype Biology. **a** Immunostained microscopy images of RestCAFs and ProCAFs cultured in 2D. Blue = DAPI; Red = F-Actin; Green = Vimentin; Magenta = α-SMA. **b**,** c** Secreted Factor Analysis of CAF conditioned media. **b** Bar chart comparing the concentration of each target expressed in RestCAF and ProCAF conditioned media. *n* = 3 for all samples. All concentrations were normalized compared to CAF medium. Unpaired T-tests were corrected for multiple comparisons with the Holm-Šídák method to determine statistical significance between RestCAF and ProCAF expression levels. * = *p* < 0.05. **c** Threshold chart illustrating the RestCAF/ProCAF expression ratio of each secreted factor. The Y axis denotes the fold-change comparison between expression levels in RestCAF compared to ProCAF. Values above 1 are expressed more highly in RestCAF, and values below 1 are expressed more highly in ProCAF

To confirm mesenchymal lineage and establish the expression of fibroblast markers by the RestCAF and ProCAF lines, we first utilized immunofluorescent staining to identify the presence of vimentin and alpha-smooth muscle actin (α-SMA) in RestCAFs and ProCAFs in 2D. Vimentin is a cytoskeletal protein found in all mesenchymal cells, and α-SMA is expressed in myofibroblasts, occasionally used as a pan-CAF marker. Maximum projection images for both RestCAFs and ProCAFs illustrate expression of vimentin (green) and α-SMA (magenta, lower images), in addition to DAPI (cell nucleus, blue) and F-Actin (cytoskeleton, red). (Fig. 1a).

Since CAFs are well known to produce a diverse secretome that can augment growth, immunogenicity, and cellular crosstalk in the TME [39], understanding the secreted factors produced by RestCAFs and ProCAFs could provide clues into the biological mechanisms these novel subtypes mediate. Thus, we performed a multiplex immunoassay and a TGF-β1 ELISA on conditioned media (CM) from RestCAFs and ProCAFs to identify differences in secreted factor production. CAF conditioned media was harvested from 2D CAF cultures after 72 hours of culture. For this experiment, we selected secreted factors that have roles in angiogenesis and immune cell recruitment. (Fig. 1b). The analysis resulted in the identification of 3 secreted factors significantly upregulated in ProCAFs compared to RestCAFs: stromal cell derived factor 1 alpha (SDF-1α, 14,571 ± 1,540.5 pg/mL vs. 342.8 ± 25.13 pg/mL, *p* = 0.0008), monocyte chemoattractant protein 1 (MCP-1, 986.2 ± 10.46 pg/mL vs. 672.9 ± 14.93 pg/mL, *p* = 0.0001), and granulocyte colony stimulating factor (G-CSF, 4.609 ± 1.126 pg/mL vs. 0.8316 ± 0.1760 pg/mL, *p* = 0.032). Additionally, 4 secreted factors were significantly upregulated in RestCAFs compared to ProCAFs: interleukin-8 (IL-8, 2,056.0 ± 45.54 pg/mL vs. 83.73 ± 4.152 pg/mL, *p* < 0.0001), transforming growth factor beta 1 (TGF-β1, 1,698.0 ± 86.54 pg/mL vs. 400.1 ± 309.8 pg/mL, *p* = 0.017), hepatocyte growth factor (HGF, 1,091.0 ± 23.60 pg/mL vs. 56.38 ± 9.566 pg/mL, *p* < 0.0001), and interferon-gamma-inducible protein 10 (IP-10, 4.391 ± 0.2291 pg/mL vs. 1.638 ± 0.1522 pg/mL, *p* = 0.0006). Of the proteins for which we detected significant differences in concentrations produced by RestCAF and ProCAF, calculating the fold change identifies two secreted factors heavily overexpressed by each CAF subtype (Fig. 1c). The concentration of SDF-1α was 42-fold greater for ProCAFs, and the concentration of IL-8 was 24-fold higher for RestCAFs. SDF-1α is a protein that has been shown to induce immunosuppression and promote angiogenesis in PDAC [40, 41], and IL-8 is the primary chemoattractant of neutrophils [42]. Concentrations of C-C motif chemokine ligand 5 (CCL5), epidermal growth factor (EGF), fibroblast growth factor-2 (FGF-2), platelet derived growth factor-BB (PDGF-BB), and tumor necrosis factor-α (TNF-α) were undetectable or negligible in supernatant from both CAF subtypes. Of note, the concentrations of vascular endothelial growth factor (VEGF-A) and interleukin-6 (IL-6) were very high (> 5,000 pg/ml) for both CAF subtypes, although no significant differences were found between the concentrations produced by RestCAFs and ProCAFs. VEGF stimulates endothelial growth and angiogenesis and is associated with leaky, dysfunctional tumor vasculature [43]. IL-6 was also abundant, possibly contributing to angiogenesis, leukocyte recruitment, B cell differentiation, and fibroblast contractility in some cancers [44–46]. Overall, these results support the presence of heterogeneous secreted factor profiles between the RestCAF and ProCAF lines tested and lays important groundwork for our investigation into unique functional differences between these novel CAF subtypes in 3D microfluidic devices.

### RestCAF line induces the formation of perfusable self-assembled microvascular networks

**Fig. 2:**
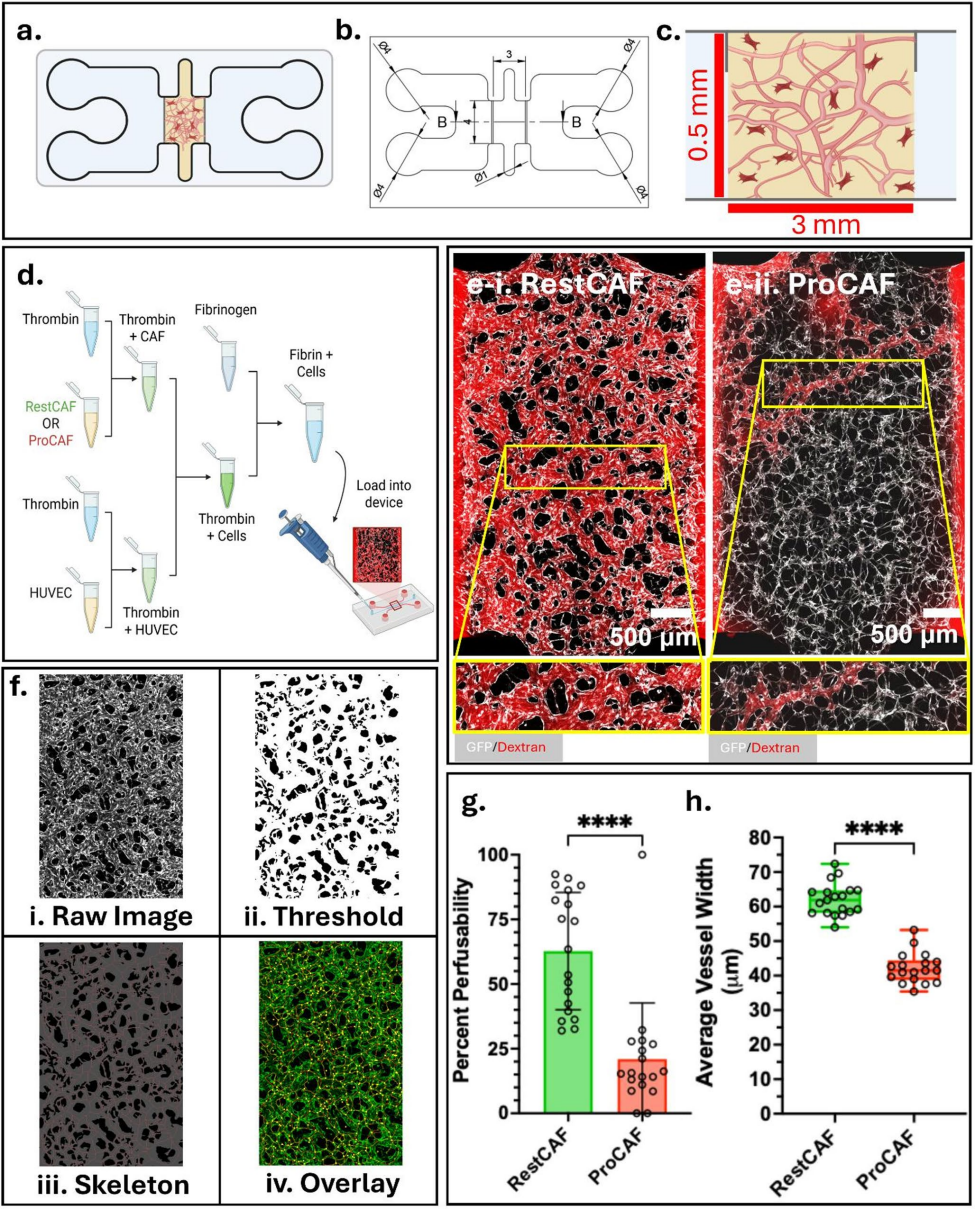
RestCAF and ProCAF-mediated self-assembling vasculogenesis of HUVECs. **a** Schematic of a three-channel microfluidic device, showing center culture region flanked by two media channels. The center channel was filled with a suspension of RestCAFs/HUVECs or ProCAFs/HUVECs in a fibrin ECM. The outer media channels were filled with Vasculife culture media. **b** CAD drawing of device. Key dimensions and diameters include four 4 mm diameter media inlet ports, two 1 mm ECM inlet ports, and a 3 mm x 4 mm x 0.5 mm center channel volume for MVN assembly in 3D. **c** Cross-section of center culture region, showing the 3 mm width and 0.5 mm height of the channel. **d** Experimental workflow schematic, showing how the combination of HUVEC, CAF, thrombin, and fibrinogen are seeded into the center channel of the device. **e** Representative images of MVNs formed with RestCAF **e-i** and ProCAF **e-ii** after 7 days of culture. White = GFP HUVEC, Red = 70 kDa dextran. Inset images highlight the perfusability and vessel diameter differences between CAF types. **f** Overview of the analysis pipeline for the Matlab-based AutoTube vessel analysis software. Raw image files **(i)** undergo thresholding to define vessel structures and the MVN area **(ii)** and skeletonization to define the MVN length **(iii)**. An overlay image of the MVN, skeletonization, and branching points is also shown **(iv)**. Representative images are GFP-HUVEC MVNs formed with RestCAFs. **g** Bar chart showing the MVN perfusability percentage for each RestCAF and ProCAF device. Bars display mean, whiskers display SD, and each data point represents the percent perfusion within a single device. A two-sample T-test was used to determine statistical significance. RestCAF *n* = 19 devices, ProCAF *n* = 18 devices. **** = *p* < 0.0001. **h** Boxplot showing the average MVN vessel width within each RestCAF and ProCAF device. Each data point represents the average MVN vessel width value within a single device, and whiskers illustrate the minimum and maximum average widths. A two-sample T-test was used to determine statistical significance. RestCAF *n* = 19 devices, ProCAF *n* = 18 devices. **** = *p* < 0.0001

Vasculogenesis is the biological process of *de novo* blood vessel formation. The formation of branching, perfusable microvascular networks (MVNs) within microfluidic devices is an example of this process, and is a tissue engineering method often used to create 3D models suitable for testing vascular functions, such as blood flow, permeability, and extravasation [35]. Stromal cells, like fibroblasts, are instrumental in regulating vasculogenesis, due to the presence of cell-derived contractile forces often being necessary for open, perfusable lumen formation [47]. Therefore, to better understand the ability for RestCAFs and ProCAFs to mediate vasculogenesis, we utilized a three-channel microfluidic device to observe CAF-driven MVN formation (Fig. 2a). The microfluidic device contains a central cell culture channel flanked by two media channels, enabling MVN formation in 3D (Fig. 2b and c). To generate self-assembled MVNs, RestCAFs or ProCAFs were mixed with HUVECs in a fibrin ECM and injected into the center channel of the microfluidic device (Fig. 2d). After MVN self-assembly over 7 days of incubation, we flowed 70 kDa fluorescent dextran through the network to identify perfusable vessels with open lumens. Confocal microscopy allowed us to visually observe clear differences in MVN morphology, with many vessels formed in RestCAF devices perfusable to dextran (Fig. 2e-i). Conversely, most of the vessels formed in ProCAF devices did not open into perfusable lumens, with a representative image illustrating one perfusable conduit surrounded by a network of narrow endothelial structures lacking open lumens (Fig. 2e-ii). These results hint at potential differences in cytokine signaling between RestCAFs and ProCAFs that may explain the observed differences in successful vasculogenesis leading to perfused vessels.

To quantify perfusion and geometry of MVNs formed with RestCAFs and ProCAFs and validate our visual observations, we utilized the AutoTube vessel geometry quantification software within Matlab [38]. After capturing maximum projection confocal microscopy images of GFP-HUVEC MVNs and perfusing fluorescent dextran to show open lumens, each image was loaded into AutoTube for analysis. We used the software to create a binary mask over identified vessels (used to calculate vessel area within an image) and generated morphological skeletons for the calculation of total vessel length (Fig. 2f). To calculate percent perfused in each device, the vessel area of the endothelial channel was divided by the vessel area of the dextran channel. Average vessel width values were calculated by dividing the vessel areas by the skeletonization length for each image. We identified significantly less dextran perfusion within MVNs formed with ProCAFs, compared to MVNs formed with RestCAFs, confirming our visual observations (Fig. 2g). Within ProCAF devices, the average perfusion percentage was 20.96% ± 21.75%, compared to RestCAF devices with an average of 62.74% ± 22.69% (*p* < 0.0001). Furthermore, the widths of ProCAF MVNs were significantly smaller compared to RestCAF MVNs, which could explain the low perfusion of ProCAF devices. RestCAF devices had an average vessel width of 62.06 ± 4.695 μm, compared to 42.20 ± 4.450 μm within ProCAF devices (*p* < 0.0001). Overall, these results confirm the differences in MVN geometry observed visually and demonstrate the opposing effects of these RestCAF and ProCAF lines on vasculogenesis.

### RestCAF line induces enhanced extracellular matrix remodeling compared to ProCAF line

**Fig. 3:**
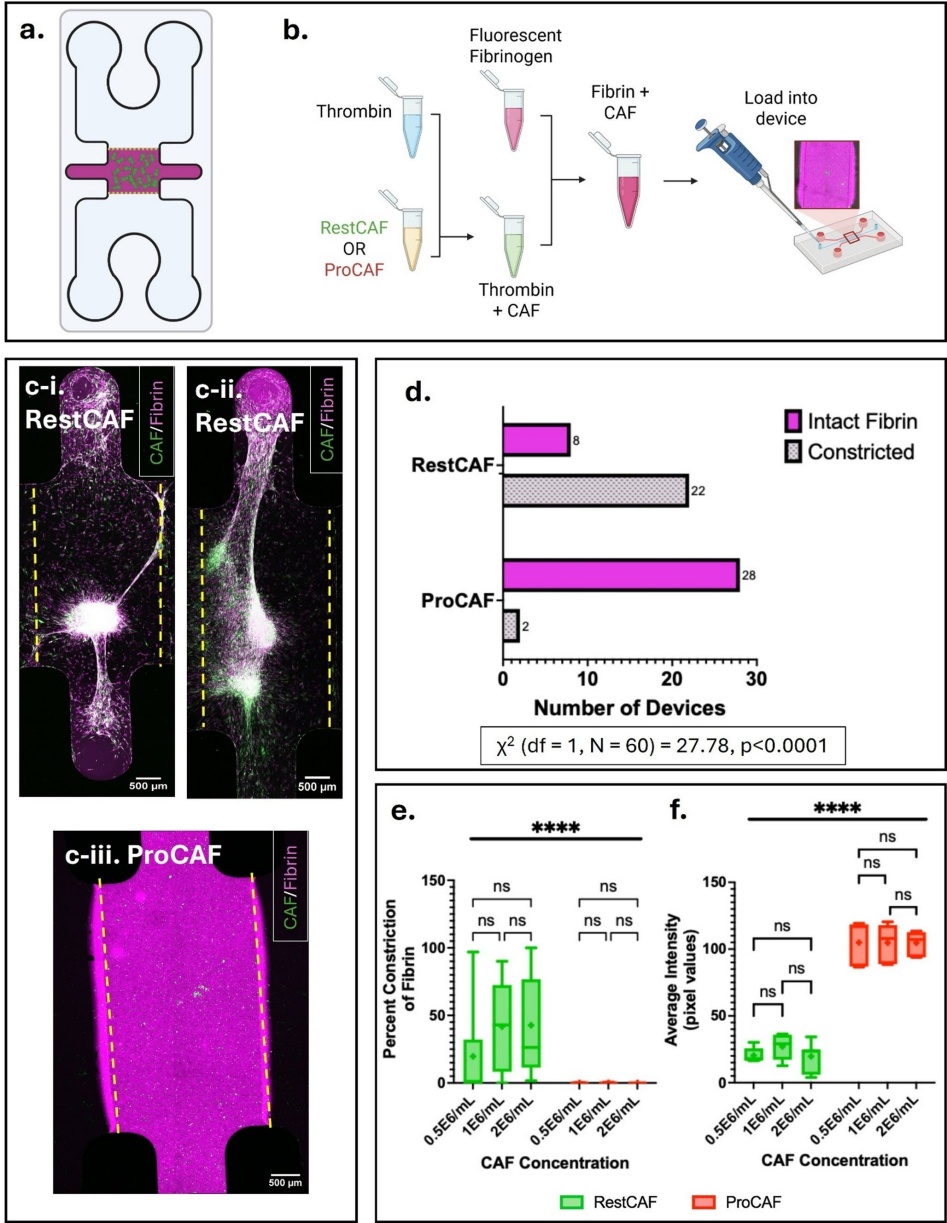
Quantifying matrix remodeling properties of RestCAF and ProCAF. **a** Schematic of three-channel microfluidic device, with center channel filled with fibrin (magenta) and RestCAF/ProCAF (green). The outer media channels were filled with Vasculife culture media. **b** Experimental workflow schematic, showing how the combination of CAF, thrombin, and fibrinogen are seeded into the center channel of the device. **c** Maximum Projection confocal images of center cell region of microfluidic devices after 7 days of culture, showing opposing constriction of CAFs (green) and fibrin (magenta). RestCAF devices show large areas of fibrin constriction, forming large masses of cells and fibrin in white **(c-i**,** c-ii).** ProCAF devices did not exhibit this phenotype, with the fibrin remaining uniformly spread within the center channel **(c-iii)**.** d** Bar chart showing the number of devices that exhibited fibrin constriction and the number of devices with intact fibrin after 7 days of culture for RestCAF devices and ProCAF devices. A Chi-Square Test of Independence confirms a significant relationship between CAF subtype and constriction ability. *n* = 30 devices for both CAF types. **e** Box plot showing the percentage of fibrin constriction within each device after 7 days of culture, across CAF concentrations of 0.5$\:\times\:$10^6^, 1$\:\times\:$10^6^, and 2$\:\times\:$10^6^ cells/mL. A Student’s T test was used to determine statistical significance across CAF groups, and a Two-Way ANOVA was used to determine statistical significance between concentrations. *n* = 30 devices for both CAF types. **** = *p* < 0.0001. **f** Box plot showing the average pixel intensity of fluorescent fibrin measured after 7 days of culture, across CAF concentrations of of 0.5$\:\times\:$10^6^, 1$\:\times\:$10^6^, and 2$\:\times\:$10^6^ cells/mL. A Student’s T test was used to determine statistical significance across CAF groups, and a Two-Way ANOVA was used to determine statistical significance between concentrations. *n* = 30 devices for both CAF types. **** = *p* < 0.0001

Our CAF-driven vasculogenesis experiments resulted in the identification of distinct perfusion differences in vasculogenesis induced by the RestCAF and ProCAF lines tested. Due to the lack of perfusable MVNs formed by these ProCAFs, we further hypothesized that these RestCAF and ProCAF lines may exhibit differential contractile properties within a fibrin ECM, since fibroblasts are well known to be involved in ECM remodeling, and contractile forces within the stroma may be required for open vascular lumen formation [47]. Using the same 3-channel microfluidic platform (Fig. 3a), RestCAFs and ProCAFs were embedded in a fluorescent fibrin ECM at three concentrations (0.5$\:\times\:$10^6^, 1$\:\times\:$10^6^, and 2$\:\times\:$10^6^ cells/mL) (Fig. 3b) and imaged after 7 days of culture (consistent with vasculogenesis experimental duration). In devices with RestCAF, we frequently observed the detachment of fibrin from the walls of the device, forming bundles of cells and fibrin (Fig. 3c-i, c-ii). On the other hand, devices with ProCAF predominantly did not result in the constriction or detachment of fibrin regions, with the center microfluidic channel remaining filled with fluorescent fibrin embedded with ProCAFs homogeneously (Fig. 3c-iii). To quantify these observations, we classified 30 images of RestCAF/Fibrin devices and 30 images of ProCAF/Fibrin devices into “Constricted” and “Intact Fibrin” groups, based on the presence or absence of fibrin constriction within a device, respectively (Fig. 3d). Interestingly, we observed 22 RestCAF devices exhibiting fibrin constriction compared to only 2 ProCAF devices, suggesting the presence of a significant difference in contractility mechanisms between our tested RestCAF and ProCAF lines. Likewise, 28 ProCAF devices had fully intact fibrin hydrogels, compared to just 8 RestCAF devices (*p* < 0.0001).

Furthermore, to determine differences in the magnitude of fibrin constriction between RestCAFs and ProCAFs, we calculated the percentage of fibrin constriction within a device compared to the original surface area of fibrin within the center device channel. We show that RestCAFs exhibit greater magnitudes of fibrin constriction, with a 34.71% ± 37.01% average reduction in fibrin area. In comparison, ProCAFs demonstrated a 0.031% ± 0.121% average reduction in fibrin area, demonstrating significantly greater fibrin constriction by RestCAFs (*p* < 0.0001). Notably, this effect was independent of CAF concentration, as no significant differences were found between concentration groups of 0.5$\:\times\:$10^6^, 1$\:\times\:$10^6^, and 2$\:\times\:$10^6^ cells/mL (Fig. 3e). Additionally, we analyzed the fluorescent intensity of fibrin within every device to confirm observations of degraded fluorescent fibrin intensity within RestCAF devices. Indeed, the mean pixel intensity of fluorescent fibrin within RestCAF devices was 22.35 ± 9.090, compared to a mean pixel intensity of 104.8 ± 13.34 within ProCAF devices – a statistically significant difference between CAF subtypes (*p* < 0.0001). Again, varying CAF concentration did not influence fibrin intensity values (Fig. 3f). In summary, these results suggest that the RestCAF line tested is more actively involved with ECM remodeling, and future work could examine whether they may contribute to tumor control through contractile mechanisms such as alignment of ECM fibers or through enzymatic degradation and secretion of ECM.

### Establishing a perfusable single-vessel MPS model surrounded by CAFs

**Fig. 4:**
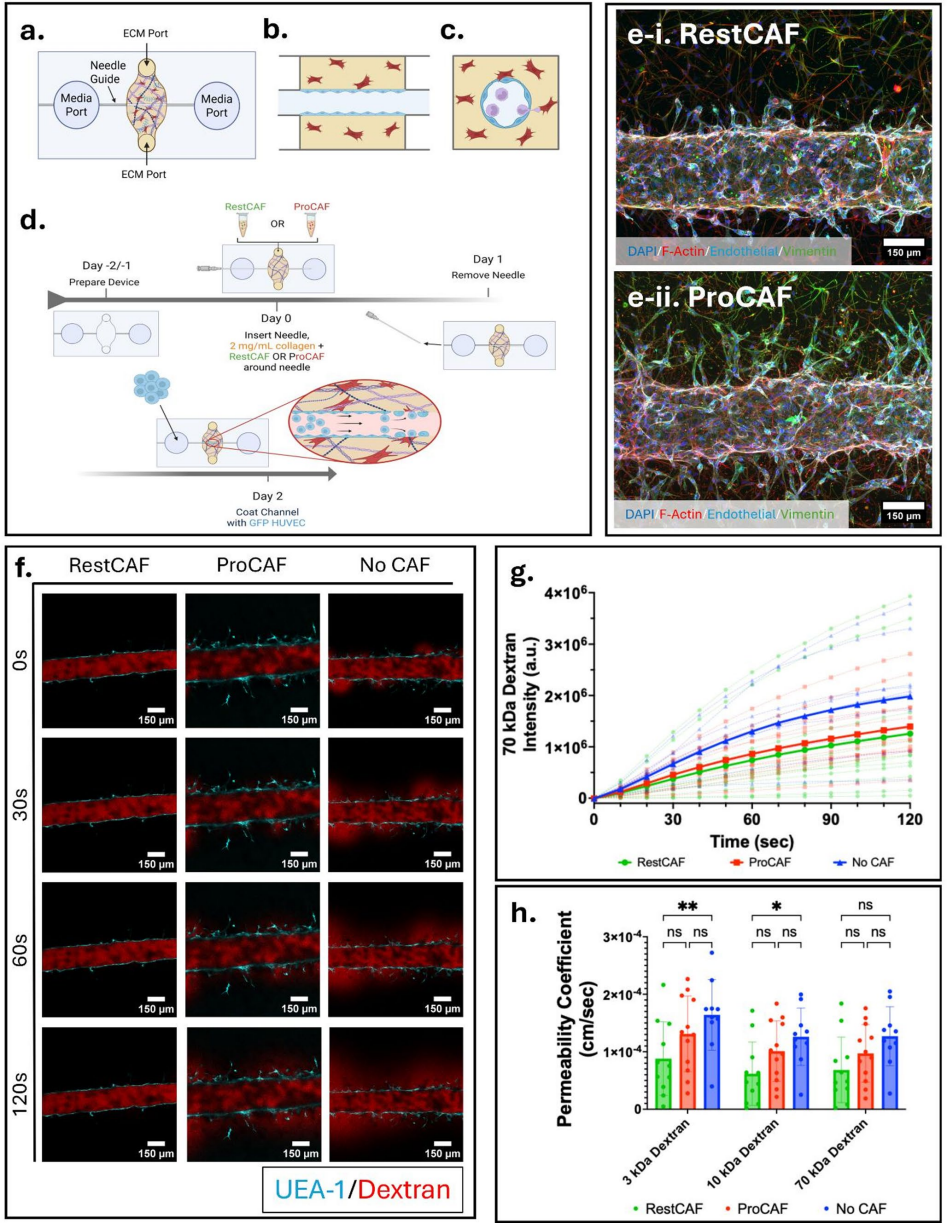
Validation of the single-vessel microfluidic device platform. **a** Microfluidic device schematic, illustrating the location of the ECM region, media ports, and needle guide. **b** An orthogonal view schematic shows the bisection of the collagen/CAF region by the HUVEC-lined vessel, formed by the polymerization of collagen around a stainless-steel needle. **c** A parallel view schematic shows the circular cross-section of the vessel, with HUVECs coated circumferentially around the vessel lumen. **d** Experimental timeline schematic, showing collagen seeding, needle removal, and HUVEC attachment to the channel wall. **e** Immunostained maximum projection images of single-vessel devices with RestCAF (**e-i**) and ProCAF (**e-ii**). Blue = DAPI; Red = F Actin; Cyan = Endothelial (UEA1+HUVEC); Green = Vimentin. **f** Time-series images of vessel permeability in RestCAF, ProCAF and No CAF control devices. Vessels were filled with 70 kDa dextran, and images were captured every 10 seconds at the midplane of the vessel for 2 minutes. Cyan = UEA1+HUVEC; Red = 70 kDa dextran. **g** Intensity of 70 kDa dextran outside of the vessel over time. Dextran intensity values over time for each device are connected with lines; mean intensity values over time across conditions are bolded. RestCAF: n = 11 devices, ProCAF: n = 12 devices, No CAF: n = 10 devices. **h** Bar plot displaying permeability coefficients for each CAF type and for each dextran weight. Each data point represents the permeability coefficient calculated from a single device. A Two-Way ANOVA and Tukey’s HSD post-hoc tests were used to determine statistical significance. RestCAF: n = 11 devices, ProCAF: n = 12 devices, No CAF: n = 10 devices. * = *p*<0.03, ** = *p*<0.002

Since the ProCAF line used in this study did not support the formation of perfusable MVNs, an alternative model was required to analyze CAF-dependent changes in vascular functions and immune recruitment with greater control and reproducibility. Thus, we adapted a microfluidic platform from Polacheck et.al [34] to generate a micropatterned blood vessel cast from a stainless-steel needle. The vessel lumen was fabricated by inserting the needle through the microfluidic device, through a small needle guide (Fig. 4a). This needle bisects a central ECM chamber, which was filled with CAFs embedded in type-1 collagen (Fig. 4b). After collagen polymerization, the needle was removed, and the hollow lumen was lined with HUVECs (Fig. 4c). This results in a cylindrical, endothelial-lined blood vessel surrounded by ECM containing CAFs (Fig. 4c, d). We fixed and stained several devices 2 days after introducing HUVECs to demonstrate the uniform endothelial monolayer (cyan) surrounded by RestCAF (Fig. 4e-i) and ProCAF (Fig. 4e-ii) stained for the mesenchymal marker vimentin (green), with all cells indicated with counterstains for the nucleus (DAPI, blue) and F-actin (phalloidin, red).

To determine the effects of RestCAFs and ProCAFs on vascular barrier function, we evaluated vessel permeability 1 day after coating the lumen with HUVECs. Three molecular weights of fluorescent dextran (3 kilodalton (kDa), 10 kDa, and 70 kDa) were flowed through the vessel for 2 minutes, with images captured at the midplane of the vessel every 10 seconds to observe leakage of the dye through time. These dextran sizes are expected to diffuse paracellularly across the endothelium [48, 49]. Importantly, 70 kDa dextran is approximately similar in size to human albumin – a protein that predominantly stays within the bloodstream – thus serving as a suitable marker for physiological permeability values of our single-vessel model. Visually, we observed less leakage of 70 kDa dextran in devices containing RestCAF than those with ProCAFs or lacking CAF, with time-series images from representative devices highlighting 70 kDa dextran leakage from the vessel into collagen over time (Fig. 4f).

Quantifying dextran intensity in collagen over time and calculating characteristic permeability coefficients was performed with Matlab code provided by Polacheck et al [34]. This code defines a vessel region and an ECM region based on initial fluorescent dextran intensity values, and computes both dextran intensity over time and permeability coefficients. Plotting 70 kDa dextran intensity in the collagen region through time allowed us to compare the leakage of dye through the vessel wall into the ECM, where a large spread of 70 kDa dextran intensity values across RestCAF, ProCAF, and control devices was noted (Fig. 4g). Indeed, permeability coefficient values for each device had high degrees of variability between devices. Despite this variability, we identified significantly lower permeability in devices with RestCAF compared to control devices for 3 kDa dextran (*p*=0.0079) and 10 kDa dextran (*p*=0.0296) (Fig. 4h). Although visible data trends appear to suggest that permeability is lower in RestCAF devices than in ProCAF devices, we did not observe statistically significant differences in vessel permeability between devices with RestCAFs and ProCAFs across molecular weights of dextran. The high variability could be due to the presence of holes in the endothelial monolayer as a result of inconsistent monolayer formation during cell seeding, which is a limitation of endothelial channel coating-based microfluidic models as a whole [34]. Nevertheless, this single-vessel model allows for creating a 3D, perfusable, vascularized model to ultimately assess the immunogenicity of RestCAFs and ProCAFs with greater control and reproducibility.

### RestCAFs and ProCAFs induce changes in sprouting angiogenesis and vessel geometry

**Fig. 5:**
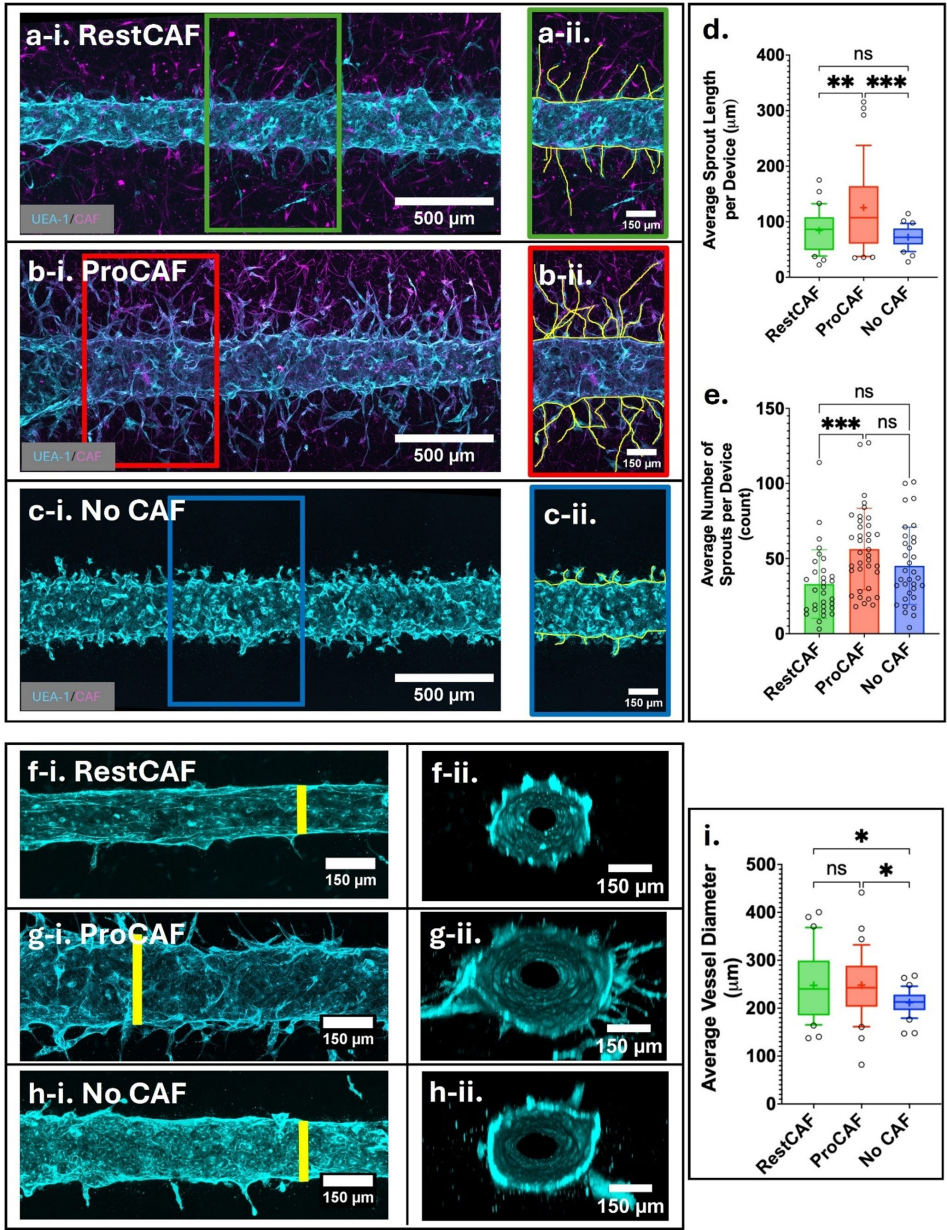
CAF-induced changes in sprouting angiogenesis and vessel geometry. **a**,** b**, **c** Representative maximum projection confocal images of angiogenic sprouting in devices with RestCAF **(a-i)**, ProCAF **(b-i)**, and No CAF control devices **(c-i)**. Image insets clearly show angiogenic sprout length measurements used for calculations in yellow, in devices with RestCAF **(a-ii)**, ProCAF **(b-ii)**, and No CAF **(c-ii)**. **d)** Box plot displaying the average angiogenic sprout lengths within each device, for each CAF type. Whiskers cover 10%-90% of values. A One-Way ANOVA with Tukey’s HSD post-hoc tests were used to determine statistical significance. RestCAF *n* = 31 devices, ProCAF *n* = 37 devices, No CAF *n* = 33 devices. **=*p* < 0.0021, ***=*p* < 0.0002. **e** Bar chart displaying average numbers of sprouts within one device, for each CAF type. Each data point represents the average sprout length within one device, and whiskers cover 5%-95% of values. A One-Way ANOVA with Tukey’s HSD post-hoc tests were used to determine statistical significance. RestCAF *n* = 31 devices, ProCAF *n* = 37 devices, No CAF *n* = 33 devices. ***=*p* < 0.0002. **f**,** g**, **h** Representative maximum projection confocal images demonstrating changes in vessel diameter within RestCAF **(f-i)**, ProCAF **(g-i)**, and No CAF devices **(h-i)**. Yellow lines illustrate example vessel diameter measurement locations. 3D cross-section images for RestCAF **(f-ii)**, ProCAF **(g-ii)**, and No CAF devices **(h-ii)** further illustrate differences in vessel diameters between CAF types. **i)** Box plot showing average diameters of vessels for each CAF type. Each data point represents the average vessel diameter within a single device, and whiskers cover 10%-90% of values. A One-Way ANOVA with Tukey’s HSD post-hoc tests were used to determine statistical significance. RestCAF *n* = 31 devices, ProCAF *n* = 37 devices, No CAF *n* = 33 devices. *=*p* < 0.05

Throughout the process of optimizing cell culture parameters within the single-vessel model, we observed several differences in how CAFs altered the geometry of the vessel within this platform, including changes in sprouting angiogenesis and the diameter of the main HUVEC-coated vessel. Angiogenesis is the formation of new blood vessels that extend from existing vasculature, and this process often serves as a key hallmark of tumor progression [50]. To this end, we measured the lengths of observed angiogenic sprouts and the diameter of endothelial vessels within the single-vessel model after 2 days of co-culture. Representative maximum projection confocal images illustrate the formation of long, narrow, angiogenic sprouts in both RestCAF devices (Fig. 5a-i) and ProCAF devices (Fig. 5b-i). On the other hand, control devices lacking CAFs largely contained short, stubby sprout formations (Fig. 5c-i). These images suggest that this ProCAF line stimulates longer and more numerous angiogenic sprouts. To confirm our visual observations, we quantified sprout lengths by tracing their paths from maximum intensity projections of confocal images (yellow lines, Fig. 5a-ii, b-ii, c-ii). Indeed, after measuring the length of each sprout and calculating the average sprout length within each device, we determined that ProCAFs induced significantly longer angiogenic sprouts than RestCAFs or control devices without CAFs (Fig. 5d). ProCAFs stimulated a mean sprout length of 125.3 ± 78.25 μm, compared to 84.11 ± 38.15 μm for RestCAFs (*p* = 0.0055) and 72.13 ± 19.64 μm for control devices (*p* = 0.0002). Furthermore, the presence of ProCAFs also increased the number of sprouts within a device (Fig. 5e). The average number of sprouts within ProCAF devices was 56.30 ± 27.11, which was significantly greater than RestCAF devices (33.00 ± 22.84, *p* = 0.0008), but not significantly different compared to control devices lacking CAF (45.15 ± 25.78, *p* = 0.1651). Thus, these results suggest the prominent role of ProCAFs in moderating angiogenic sprouting compared to the RestCAFs, and hints at the potential role of ProCAFs in mediating a clinically impactful response of vasculature in the TME.

We also observed notable changes in the diameter of the main endothelial vessel across CAF conditions during the 2 days of culture. Similar to angiogenesis, changes in vessel diameter through vasodilation could also serve as a promotor for tumor progression [51, 52]. To determine if there was a CAF-mediated influence on vessel diameter, three diameter measurements were recorded along the length of each vessel, including the minimum, maximum, and average vessel diameter, determined visually. All vessels began with a diameter of 160 μm, the size of the needle used to cast each channel. Representative images display examples of one measurement location for each condition (yellow bars, Fig. 5f-i, g-i and h-i), and corresponding 3D cross-section images highlight changes in vessel diameters within RestCAF devices (Fig. 5f-ii), ProCAF devices (Fig. 5g-ii), and control devices (Fig. 5h-ii). The was no significant difference detected between the average vessel diameters of RestCAF devices (247.5 ± 74.13 μm) and ProCAF devices (248.0 ± 68.08 μm, *p* = 0.9995). However, vessels in RestCAF devices and ProCAF devices were both significantly increased compared to control vessels (211.6 ± 26.65 μm, RestCAF/control *p* = 0.0487, ProCAF/control *p* = 0.0347) (Fig. 5i). Interestingly, we noticed a much larger range of vessel diameters within CAF-containing devices compared to control devices, suggesting that the presence of CAFs results in complex intercellular interactions such as endothelial cell proliferation, ECM remodeling, or localized cell contractility.

### RestCAF and ProCAF lines induce differential recruitment of immune cells

**Fig. 6:**
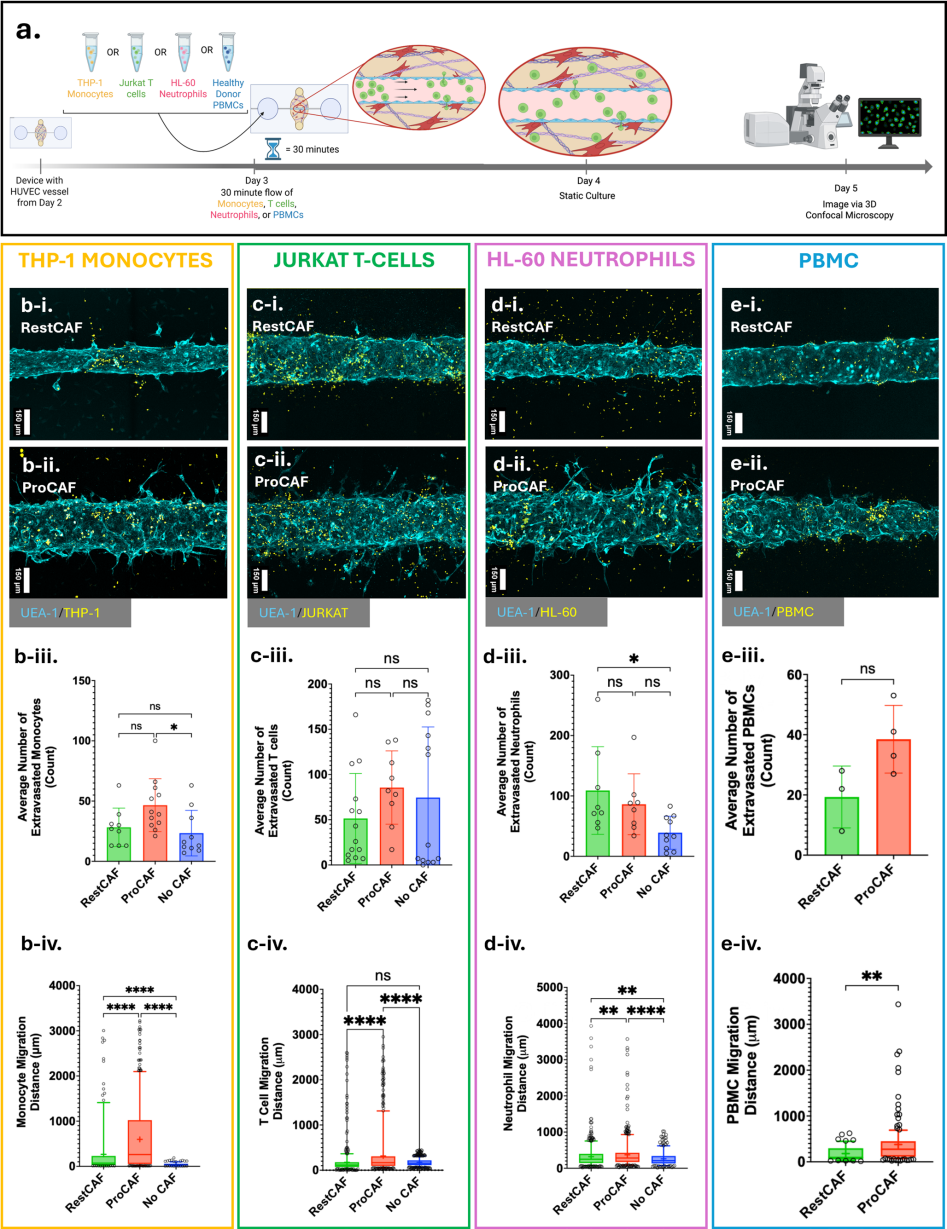
Assessing CAF-mediated immune cell recruitment. **a** Experimental timeline schematic, showing immune cell flow through HUVEC tube, static culture/migration, and imaging. **b**,** c**, **d**,** e** Immune cell recruitment results for **b** THP-1 monocytes, **c** Jurkat T cells, **d** HL-60 derived neutrophils, and **e** primary human Peripheral Blood Mononuclear Cells (PBMC). For each immune cell type, a maximum projection confocal microscope image shows migrating immune cells (yellow) within RestCAF devices **(i)** or ProCAF devices **(ii)**. All statistical tests utilized were One-Way ANOVA with Tukey’s HSD post-hoc tests. **b-iii** Average number of extravasated monocytes per device for RestCAF, ProCAF, and No CAF control devices. Bars display means, whiskers display SD, and each data point represents the mean value of extravasated monocytes in one device. RestCAF *n* = 9 devices, ProCAF *n* = 12 devices, No CAF *n* = 10 devices. *=*p* < 0.05. **b-iv** Migration distance (µm) of THP-1 monocytes into the ECM from the endothelial barrier for RestCAF, ProCAF, and No CAF control devices. Each data point represents the migration distance of one monocyte, and whiskers cover 5%-95% of migration distances. RestCAF *n* = 254 cells, ProCAF *n* = 557 cells, No CAF *n* = 234 cells. ****=*p* < 0.0001. **c-iii** Average number of extravasated T cells per device for RestCAF, ProCAF, and No CAF control devices. Bars display means, whiskers display SD, and each data point represents the mean value of extravasated T cells in one device. RestCAF *n* = 14 devices, ProCAF *n* = 9 devices, No CAF *n* = 13 devices. **c-iv** Migration distance (µm) of T cells into the ECM from the endothelial barrier for RestCAF, ProCAF, and No CAF control devices. Each data point represents the migration distance of one T cell, and whiskers cover 5%-95% of migration distances. RestCAF *n* = 719 cells, ProCAF *n* = 759 cells, No CAF *n* = 968 cells. ****=*p* < 0.0001. **d-iii** Average number of extravasated neutrophils per device for RestCAF, ProCAF, and No CAF control devices. Bars display means, whiskers display SD, and each data point represents the mean value of extravasated neutrophils in one device. RestCAF *n* = 8 devices, ProCAF *n* = 8 devices, No CAF *n* = 10 devices. *=*p* < 0.05. **d-iv** Migration distance (µm) of neutrophils into the ECM from the endothelial barrier for RestCAF, ProCAF, and No CAF control devices. Each data point represents the migration distance of one neutrophil, and whiskers cover 5%-95% of migration distances. RestCAF *n* = 819 cells, ProCAF *n* = 686 cells, No CAF *n* = 391 cells. **=*p* < 0.002. ****=*p* < 0.0001. **e-iii** Average number of extravasated primary human PBMCs per device for RestCAF and ProCAF devices. Bars display means, whiskers display SD, and each data point represents the average number of extravasated PBMCs in one device. RestCAF *n* = 3 devices, ProCAF *n* = 4 devices. **e-iv** Migration distance (µm) of PBMCs into the ECM from the endothelial barrier for RestCAF and ProCAF devices. Each data point represents the migration distance of one PBMC, and whiskers cover 5%-95% of migration distances. RestCAF *n* = 58 cells, ProCAF *n* = 154 cells

The ability of CAFs to influence the immune response within the PDAC TME is well studied [11, 23, 25]. Therefore, we assessed the potential of RestCAF and ProCAF lines to recruit immune cells into the TME by flowing THP-1 monocytes, Jurkat T cells, HL-60-derived neutrophils, and primary human Peripheral Blood Mononuclear Cells (PBMCs) through the single-vessel devices. After coating the channel with HUVECs and allowing a 2-day culture period for confluent monolayers to form, immune cells were flowed through the vessels to observe adhesion and extravasation through the endothelium, with the migration distance of each immune cell into the CAF-filled ECM measured after an additional 2 days of static culture (Fig. 6a). Migration distances for monocytes (Fig. 6b), T cells (Fig. 6c), neutrophils (Fig. 6d), and PBMCs (Fig. 6e) were quantified by measuring the perpendicular distance between the center of a cell (yellow spheres) and the vessel wall (cyan) in maximum intensity projection images for RestCAF devices (panel i), ProCAF devices (panel ii), and control devices without CAFs (images not shown).

To begin, we tested the extravasation of THP-1 monocytes. After quantifying both the number of extravasated monocytes and the extravasation distance from the vessel wall, we identified an average of 28.22 ± 15.98 extravasated monocytes in RestCAF devices, 46.58 ± 21.96 monocytes in ProCAF devices, and 23.40 ± 18.91 monocytes in control devices, with a significant increase identified between ProCAF devices and control devices (*p* = 0.0248) (Fig. 6b-iii). Although overall trends hint at an increased numbers of extravasated monocytes in ProCAF devices compared to RestCAF devices, the number of extravasated THP-1 monocytes between CAF types was not statistically significant (*p* = 0.0992). However, considerable differences exist between the migration distance traveled by monocytes, with the mean migration distance of monocytes in ProCAF devices (596.6 ± 718.5 μm) being significantly greater than in RestCAF devices (266.1 ± 505.7 μm, *p* < 0.0001) or control devices (42.66 ± 29.71 μm, *p* < 0.0001) (Fig. 6b-iv). Taken together, these results suggest an enhanced ability of this ProCAF line to recruit monocytes compared to the RestCAF line.

Similarly, we flowed Jurkat T cells through the single-vessel devices and quantified both the number of extravasated T cells and the migration distance of each cell. Similar to the THP-1 monocyte recruitment results, we observed no significant difference in number of extravasated T cells between RestCAF devices, ProCAF devices, and control devices (Fig. 6c-iii). On average, 51.36 ± 49.95 T cells extravasated in RestCAF devices, 85.56 ± 40.52 T cells extravasated in ProCAF devices, and 74.46 ± 78.10 T cells extravasated in control devices (RestCAF/ProCAF *p* = 0.3859, RestCAF/Control *p* = 0.5814, ProCAF/Control *p* = 0.9047). However, T cells that were able to extravasate in ProCAF devices once again migrated significantly further into the ECM compared to T cells in RestCAF and control devices (Fig. 6c-iv). The mean extravasation distance within ProCAF devices (315.3 ± 443.1 μm) was significantly greater than the mean extravasation distance in RestCAF devices (175.9 ± 292.1 μm, *p* < 0.0001) or control devices (169.9 ± 76.68 μm, *p* < 0.0001).

Next, we flowed HL-60 derived neutrophils through the single-vessel devices. Like the monocytes and T cells, we did not observe a significant difference in number of extravasated neutrophils between RestCAF devices and ProCAF devices, where an average of 109.0 ± 72.62 neutrophils extravasated in RestCAF devices compared to 86.25 ± 50.40 extravasated neutrophils in ProCAF devices (*p* = 0.6577). However, we did observe a significant increase of extravasated neutrophil numbers in RestCAF devices compared to control devices (39.10 ± 27.33 average extravasated neutrophils in control, *p* = 0.0236) (Fig. 6d-iii). This data may be correlated with secretion of IL-8 by this RestCAF line, which is worth investigating in future studies across more donors. Further, migration distance results for neutrophils largely mirror our results for monocytes and T cells, where ProCAFs induced significantly greater migration distances of neutrophils (370.7 ± 372.8 μm) compared to RestCAFs (319.4 ± 329.8 μm, *p* = 0.0061) and control devices (260.1 ± 179.1 μm, *p* < 0.0001) (Fig. 6d-iv).

Our use of the THP-1 monocyte, Jurkat T cell, and HL-60-derived neutrophil cell lines facilitates batch-to-batch reproducibility of the experimental methods for assessing CAF-driven immune cell recruitment. However, these cell lines may not exhibit all the functional behaviors of primary human immune cells. To validate our cell line-based immune recruitment findings and increase the clinical translatability of our results, we flowed PBMCs through single-vessel models, and quantified both the number of extravasated PBMCs and the migration distance in the same manner as our previous cell line experiments. The results of PBMC recruitment experiments closely mirrored those of cell lines. We observed no significant difference in number of extravasated PBMCs between RestCAF devices (19.33 ± 10.26 PBMCs) and ProCAF devices (38.50 ± 11.24 PBMCs, *p* = 0.0688), as we also observed for monocyte, T cell, and neutrophil cell lines (Fig. 6e-iii). Similarly, we observed a significant greater migration distance of PBMCs within ProCAF devices, with a migration distance of 378.5 ± 463.3 μm within ProCAF devices, compared to 181.1 ± 161.4 μm in RestCAF devices (Fig. 6e-iv). The opposing trends between extravasated cell counts and migration distance points at functional mechanistic distinctions between immune cell extravasation and chemotaxis through tissue mediated by CAFs on the broader immune cell response. The PBMC experiment provides supportive evidence in primary immune cells that is consistent with findings from immune cell lines, but additional PBMC donors and device-level statistical analysis will be needed to establish generalizability.

## Discussion

Even though CAFs are known to be highly heterogeneous, the biological mechanisms through which CAFs differentially influence tumor growth, invasion, and response to treatment are less clear. Therefore, we have developed physiologically relevant 3D microfluidic models to isolate and study how CAFs interact with the TME. Leveraging the recently identified RestCAF and ProCAF phenotypes, we can now explore the ways in which different populations of CAFs alter the TME. We identified key differences between one RestCAF cell line and one ProCAF line including secreted factors, ECM remodeling, vasculogenesis, angiogenesis, and immune cell recruitment that may facilitate future development of CAF-targeted therapies for PDAC.

One visually striking difference between the RestCAFs and ProCAFs tested was the ECM constriction and loss of fluorescent fibrin intensity we observed in microfluidic devices containing RestCAFs, whereas we did not observe appreciable fibrin constriction or remodeling in ProCAF devices. RestCAF-driven fibrin constriction resulted in large bundles of fibrin and CAFs that detached from the inner walls of the microfluidic device. These bundles may reflect the contractile forces exerted by fibroblasts [53]. These observations are especially interesting when considering the presence of α-SMA across wider ProCAF populations – a widely used marker for the highly contractile myCAF classification. Therefore, CAFs may not be able to be accurately classified into distinct subtypes based on the presence of a single marker such as α-SMA. Overall, the combination of fibrin constriction and degradation present in RestCAF devices, but not in ProCAF devices, strongly suggests the broader presence of matrix remodeling mechanisms that are activated inside this distinct RestCAF line, which is an exciting avenue for additional study. Interleukin 6 (IL-6) is one cytokine highly expressed by CAFs to investigate further, as the activation of the JAK, ROCK, and STAT3 pathways by IL-6 resulted in increased actomyosin contractility and CAF ECM remodeling in melanoma [45, 46].

Tumor vasculature is a critical component of the TME, facilitating the transport of nutrients to supply rapid tumor growth and enabling easy paths for metastasis [54]. Leaky tumor vasculature contributes to high interstitial fluid pressure in tumors, thus reducing drug transport to the tumor core, or it can facilitate the preferential accumulation of nanoparticle drugs through the enhanced permeation and retention (EPR) effect [55]. Vascular development can be classified into two major processes: vasculogenesis – the *de novo* formation of new blood vessels; and angiogenesis – the formation of new blood vessels from existing vasculature, including sprouting angiogenesis. Fibroblasts contribute instrumentally to both processes due to the production of several growth factors, especially Vascular Endothelial Growth Factor (VEGF) [50]. The microfluidic models used in this study allowed us to identify more robust stimulation of vasculogenesis by the RestCAF line compared to the ProCAF line, as indicated by the formation of perfusable vascular lumens in a self-assembly assay. Despite the high levels of fibrin constriction observed when RestCAFs were cultured alone in fibrin, RestCAFs were able to induce the formation of open, perfusable vascular networks, potentially due to the necessity of contractile forces for lumen formation [47]. Conversely, ProCAFs stimulated greater sprouting angiogenesis compared to RestCAFs, as evidenced by longer, more numerous sprouts in single-vessel microfluidic devices containing ProCAFs. Thus, these RestCAF and ProCAF lines both influence blood vessel geometry and growth, a conclusion that is reinforced by the high concentrations of VEGF produced by both CAF subtypes. Abundant production of VEGF, yet drastic differences in observed vessel phenotypes, suggests the presence of opposing vessel maturation signaling mechanisms within these CAF subtypes that enable the angiogenic phenotype of the ProCAF line and the vasculogenic phenotype of the RestCAF line. One possible explanation for the increased sprouting angiogenesis induced by ProCAFs could be the high expression of SDF-1α (CXCL12) identified in our secreted factor analysis. SDF-1α is pro-angiogenic, but has been correlated with invasion, chemoresistance, and immunosuppression in pancreatic cancer through interactions with its receptors CXCR4 and CXCR7 [40, 56]. Differences in vasculogenesis and angiogenesis may also be due to the production of additional factors, such as anti-angiogenic molecules, that we did not identify.

Since the ProCAF line did not consistently promote the formation of perfusable vasculature via self-assembly, the 3-channel microfluidic device used for vasculogenesis experiments was not a suitable platform to assess immune cell recruitment. To reproducibly enable luminal flow through blood vessels surrounded by CAFs, we adapted the model from Polacheck et al. [34] to examine the immune cell recruitment process in vitro, including flow, adherence to the endothelium, extravasation, and migration through the ECM. Using this model allowed us to identify overall trends of increased monocyte recruitment in ProCAF devices, increased neutrophil recruitment in RestCAF devices, and no notable differences in T cell recruitment in this model. Analysis of CAF conditioned media indicated increased production of the IL-8 in RestCAFs and monocyte chemoattractant protein 1 (MCP-1) in ProCAFs, which may correlate with the corresponding preferential recruitment of neutrophils and monocytes, respectively. Overall, these results suggest that the RestCAF and ProCAF lines tested contribute to distinct immune microenvironments in PDAC. It is also clear that CAFs are indeed important drivers of immune cell recruitment into the TME, but they are unlikely to be the only source of recruitment for immune cells. Additional components of the TME such as tumor cells, resident immune cells (e.g. macrophages), and ECM likely work together to define the distinct immune populations that emerge in each subtype [25], and is an important consideration for the development of future patient-specific treatments for PDAC.

The focus of this study was to utilize 3D microfluidic models of the PDAC TME that include endothelial cells and CAFs as central components. The tumor vasculature is an instrumental, yet often overlooked system that is important for successful immune recruitment [57] and drug delivery [52]. However, tumor vasculature can also support tumor growth via angiogenesis [43], facilitate hematogenous metastasis [58], produce high interstitial fluid pressure [55]. Using 3D microphysiological systems allows us to observe the dynamic vascular processes of angiogenesis and vasculogenesis in physiological conditions. Thus, these novel 3D models allow us to assess the effects that our opposing PDAC CAF subtypes have on vessel function and dynamics with high degrees of clinical translatability.

One limitation of this study is the vascular permeability measurements acquired in the single-vessel microfluidic platform. These models rarely achieve the low, physiological permeability coefficients observed in vivo or in self-assembled vasculature [59]. However, since the ProCAF line did not reliably stimulate vasculogenesis, the single-vessel model enabled perfusion of immune cells more reproducibly. In addition, we have characterized the functional effects of CAF subtypes on several features of the TME, but our analysis thus far has been limited to CAFs from two donors. Subtype identity of CAFs was also identified from isolated cells in culture prior to incorporation in microfluidic devices, and future studies can explore whether the RestCAF and ProCAF subtypes are plastic or durably retained across a range of conditions and cell combinations. In the future, we will incorporate additional donors to verify that the trends we observed in these cells extend to larger populations of RestCAFs and ProCAFs. A final limitation relates to our CAF secreted factor analysis, which relied on conditioned media derived from CAFs cultured in 2D. Our experiments primarily included CAF cultures in 3D, and secreted factor production can be different between 2D and 3D environments [60].

Incorporating PDAC organoids into the model to study how CAFs influence tumor growth, metastasis, and treatment response is a key future direction. This will allow for the creation of a complete “PDAC-on-chip” model and one that can be used to assess the efficacy of CAF-targeted treatments. Additionally, mechanistic studies could be completed to more precisely identify causes of vasculogenesis, angiogenesis, ECM remodeling, and immune recruitment differences. Inhibiting the signaling pathways of highly expressed cytokines, such as CXCR1/CXCR2 inhibition in the IL-8 pathway and CXCR4/CXCR7 inhibition of the SDF-1α pathway, could verify the importance of the CAF secretome in mediating the observed functional differences between RestCAFs and ProCAFs.

## Conclusion

In conclusion, the identification of clear functional differences between clinically prognostic PDAC CAF subtypes provides novel insight into solving the mystery of CAF heterogeneity in the TME. Our use of 3D models to mimic in vivo environments allowed us to identify dichotomous roles of two distinct RestCAF and ProCAF populations at the tissue level. The RestCAF line we tested promoted vasculogenesis and ECM remodeling, while the ProCAF line promoted angiogenesis and immune cell migration. Thus, these distinct influences of CAFs in the TME offer encouraging insights that may enable the development of novel CAF-targeted treatments for PDAC in the future.

## Supplementary Information

Below is the link to the electronic supplementary material.

Supplementary Material 1

Supplementary Material 2

## Data Availability

All data supporting the findings of this study are available within the paper and its Supplementary Information.
