# Supplementary material for: Characterizing pancreatic cancer-associated fibroblast heterogeneity in vascular microphysiological systems
Source: Angiogenesis. 2026 Jun 9;29(3):39. doi: 10.1007/s10456-026-10061-9 (PMC13249731; doi:10.1007/s10456-026-10061-9)
Supplement: Supplementary file 1 — Supplementary Material 1 [file 10456_2026_10061_MOESM1_ESM.pdf]

Supplementary Information for “Characterizing Pancreatic Cancer-Associated Fibroblast Heterogeneity in Vascular Microphysiological Systems”

Name	Excitation Peak	Brand	Catalog Number	Concentration (μM)
CellTracker Green CMFDA	492 nm	Invitrogen	C7025	5
Cell Tracker Orange CMRA	548 nm	Invitrogen	C34551	5
CellTracker Deep Red	630 nm	Invitrogen	C34565 A	1

Table S1: CellTracker Fluorescent Probe Information

Reagent	Quantity	Brand	Catalog Number
10x MEM	60 μL	Gibco	11430-030
7.5% $NaHCO_3$	36 μL	Sigma-Aldrich	S8875-500G
HEPES Buffer	10 μL	Corning	25-060-CI
3.0 mg/mL Type 1 Collagen	500 μL	Corning	354236
0.1M NaOH	18 μL	Thermo Scientific	124190010

Table S2: Collagen Recipe

Name	Molecular Weight	Excitation Wavelength	Brand	Catalog Number
Dextran, Cascade Blue™	3 kDa	400 nm	Invitrogen	D22914
Dextran, AlexaFluor™ 647	10 kDa	650 nm	Invitrogen	D7132
Dextran, Texas Red™	70 kDa	595 nm	Invitrogen	D1864

Table S3: Fluorescent Dextran Information

Host Species	Target	Fluorophore	Excitation Wavelength	Supplier	Product Number
Mouse	Human vimentin	None	None	Biolegend	677802
Goat	Mouse IgG	AlexaFluor™ 568	579 nm	Invitrogen	A11031
Goat	Rabbit IgG	AlexaFluor™ 633	631 nm	Invitrogen	A21071
Goat	Rat IgG	AlexaFluor™ 647	650 nm	Invitrogen	A21247

Table S4: Primary/Secondary Antibody Information

Name	Target	Fluorophore	Excitation Wavelength	Supplier	Product Number
DAPI	Nuclear Stain	DAPI	360 nm	Thermo Scientific	62248
Phalloidin	F-Actin	AlexaFluor™ 488	495 nm	Fisher Scientific	A12379
UEA-1 Lectin	Endothelial Cell Membrane	FITC	495 nm	Vector Laboratories	FL-1061

Table S5: Other Cellular Stains Information
